# Caffeine Reduces Reaction Time and Improves Performance in Simulated-Contest of Taekwondo

**DOI:** 10.3390/nu6020637

**Published:** 2014-02-10

**Authors:** Victor G. F. Santos, Vander R. F. Santos, Leandro J. C. Felippe, Jose W. Almeida, Rômulo Bertuzzi, Maria A. P. D. M. Kiss, Adriano E. Lima-Silva

**Affiliations:** 1 Sport Science Research Group, Department of Physical Education and Sports Science (CAV), Federal University of Pernambuco, Pernambuco 55608-680, Brazil; E-Mails: victor_will_gustavo@hotmail.com (V.G.F.S.); vander_ramon@hotmail.com (V.R.F.S.); leandrocamati@hotmail.com (L.J.C.F.); wellingtonaljr@hotmail.com (J.W.A.); 2 Endurance Performance Research Group, School of Physical Education and Sport, University of Sao Paulo, Sao Paulo 05371-140, Brazil; E-Mails: bertuzzi@usp.br (R.B.); mapedamk@usp.br (M.A.P.D.M.K.)

**Keywords:** martial arts, sport, supplementation, ergogenic aid, time and motion studies

## Abstract

The aim of this study was to investigate the effects of caffeine on reaction time during a specific taekwondo task and athletic performance during a simulated taekwondo contest. Ten taekwondo athletes ingested either 5 mg·kg^−1^ body mass caffeine or placebo and performed two combats (spaced apart by 20 min). The reaction-time test (five kicks “Bandal Tchagui”) was performed immediately prior to the first combat and immediately after the first and second combats. Caffeine improved reaction time (from 0.42 ± 0.05 to 0.37 ± 0.07 s) only prior to the first combat (*P* = 0.004). During the first combat, break times during the first two rounds were shorter in caffeine ingestion, followed by higher plasma lactate concentrations compared with placebo (*P* = 0.029 and 0.014, respectively). During the second combat, skipping-time was reduced, and relative attack times and attack/skipping ratio was increased following ingestion of caffeine during the first two rounds (all *P* < 0.05). Caffeine resulted in no change in combat intensity parameters between the first and second combat (all *P* > 0.05), but combat intensity was decreased following placebo (all *P* < 0.05). In conclusion, caffeine reduced reaction time in non-fatigued conditions and delayed fatigue during successive taekwondo combats.

## 1. Introduction

Caffeine (1,3,7-trimethylxanthine) ingestion promotes an improvement in human performance [1]. The effects of caffeine on performance are linked to both central and peripheral mechanisms. The effect of caffeine on the central nervous system (CNS) is linked to a blockade of adenosine receptors, which prevents a decrease in neuronal activity and subsequent an increase in muscle recruitment [2]. Peripherally, caffeine inhibits phosphodiesterase activity, thereby promoting increased plasma catecholamine and glycolysis activity, increasing energy availability for active muscle during exercise [1].

As a consequence of central and peripheral effects, caffeine improves performance in tasks involving psychomotor function, such as agility and decision-making accuracy [3,4,5,6]. Fosket *et al.* [7] observed that caffeine increased speed and accuracy of a football pass. Similarly, Stuart *et al.* [8] suggested that caffeine improved coordination, speed, and precision during a specific protocol measuring rugby skills. In addition, studies have also shown that caffeine enhances performance during multiple sprints, in particular during the first sprint in a set of multiple sprints [7,8,9,10,11,12]. For example, Glaister *et al.* [10] reported the effects of caffeine on a set of 12 × 30-m repeated sprints interspaced by 35-s rest periods and noted that the time to complete first three sprints were lower when caffeine was ingested compared with placebo. These results were accompanied by an increase in plasma lactate, suggesting that caffeine increased anaerobic contributions during early sprints.

These studies demonstrate that caffeine supplementation improves power, speed, agility, attention, and reaction time. All of these variables are important determinants of performance in combat sports; however, the potential benefits of caffeine supplementation on taekwondo performance have not been explored. During a typical contest, high-intensity bouts of kicking and punching are followed by long balanced periods of low-intensity effort [13,14,15,16]. Despite attack moments representing a small proportion of the contest, attacks must be performed quickly and accurately to score as many points as possible. Thus, it is plausible to suppose that caffeine could improve performance during taekwondo combats.

Therefore, the aim of this study was to investigate the effects of caffeine ingestion on performance during a taekwondo-specific reaction-time test and a simulated taekwondo contest. We hypothesized that supplementation with caffeine could reduce reaction times and increase the number of attacks and points scored during contests.

## 2. Methods

### 2.1. Participants

Ten, male taekwondo athletes (mean ± SD: age, 24.9 ± 7.3 years; body mass, 77.2 ± 12.3 kg; height, 1.75 ± 0.06 m) with at least 7 years of experience were recruited during November and December, 2011. The participants trained more than 4 h·week^−1^ for at least 6 months prior to their participation in this study. Prior to testing, the athletes were informed of the procedures, including the possible risks involved, and they signed an informed consent form. The study was approved by the Ethics and Research Committee of the Federal University of Alagoas.

### 2.2. Design

**Figure 1:**
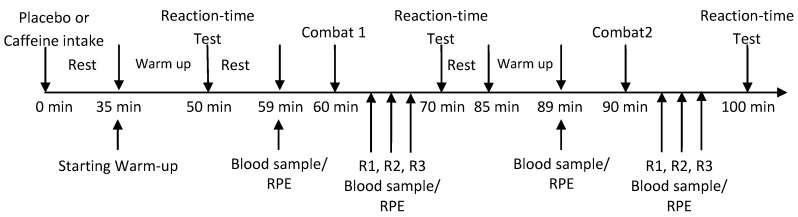
Experimental design. All experimental procedures took ~100 min. RPE: Rating of perceived effort; R1, R2, and R3: round 1, 2, and 3, respectively. Blood samples were taken to plasma lactate measurement.

During the first visit, the athletes performed an electrocardiogram to exclude any life-threatening arrhythmia or any complications arising that might cause acute problems after caffeine ingestion. During the second and third visits, the athletes consumed opaque capsules containing either caffeine (5 mg·kg^−1^ body weight) or cellulose (placebo) in a double-blind, randomized, repeated-measures crossover design (Figure 1). These 2 visits were separated by 7 days of washout. To prevent foreknowledge of treatment assignment, the sequence order (caffeine-placebo or placebo-caffeine) was implemented using a sequentially numbered container (an allocation schedule by allocation concealment to prevent selection bias and to protect the assignment sequence until allocation). Allocation and assignment for each participant was performed by an independent, blinded staff member of our laboratory. Following supplementation, the athletes remained seated for 35 min and then executed a 15-min, self-selected warm-up followed by a specific reaction-time test (Figure 1). The self-selected warm-up was recorded and repeated in the subsequent experimental session. Thereafter, a 5-min rest was allowed and the first combat was performed 1 h after supplementation. Immediately after the first combat, the athletes executed a second reaction-time test. They then remained at rest for 15 min, warmed-up again for 5 min and performed the second combat. A third reaction-time test was performed immediately after the second combat. All combats involved the same conditions (placebo *vs.* placebo and caffeine *vs.* caffeine). All procedures were concluded within 100 min after ingestion [17]. Participants were instructed to avoid alcohol, caffeine, and vigorous exercise during the 48-h period prior to each session. Participants recorded their diet 48 h before the first experimental trial and were asked to replicate it before the second experimental trial. All tests were performed at the same time of day, 4 h after the last meal to avoid any influence of circadian rhythm and previous diet on performance.

### 2.3. Reaction-Time Test

The reaction time test comprised 5 kicks interspaced by a 30-s rest period. The Bandal Tchagui, the most common technique in Taekwondo, was used [16,18]. The Bandal Tchagui is a single kick directed to the abdominal area of the opponent [19,20]. The athletes were used to performing the Bandal Tchagui several times during their regular training program. The athlete’s trochanteric height (*i.e.*, the full length of the trochanter to the floor) was measured to determine the distance to the target. An electronic device was used to emit a luminous stimulus indicating the moment that the athlete must kick the target. A movement sensor positioned in the heel of the athlete was used to determine when the athlete began to move his foot. A pressure sensor positioned in the target recorded the moment of impact. The time expended between the luminous stimulus and the movement of the foot was considered the reaction time. The reaction time was subtracted from the time expended between the luminous stimulus and foot contact with the target to retrieve the kick time. For each test, the best and worst results were excluded. The test score was defined as the mean of the three remaining kicks. The athletes performed a prior session to become familiarized with the sensors and the device, and practiced until a variation lower than 5% was found.

### 2.4. Simulated Taekwondo Contest

The simulated combats were performed in accordance with the 2009 World Taekwondo Federation’s (WTF) official rules and followed the Olympic weight categories. The fights were comprised of three 2-min rounds spaced with 1-min rest periods. The athletes were used to perform simulated combats during their regular training program, but they were not used to consume caffeine supplementation during the training sessions or competitions. The matches were refereed by a black belt in taekwondo who was blinded to the supplement that the athletes had ingested. The participants used head gear and a body protector and could see the points scored throughout the combat as in real competitions. Water was offered *ad libitum*. A video camera (Kodak Esasy Share z981, Rochester, NY, USA) was positioned at the corner of the mat, and the combats were recorded at 30 Hz for later off-line analyses.

### 2.5. Measurements

Blood samples (25 µL) were obtained from the ear lobe prior to the first and second combats, and immediately after each round to determine plasma lactate concentrations using spectrophotometry (kit Biotecnica, Varginha, Brazil; Quimis^®^, São Paulo, Brazil). Heart rate (HR) was registered continuously using a chest-mounted heart rate monitor (Polar, Vantage XL, Woodbury, NY, USA) and was stored in a computer for later analyses (Polar^®^ Heart Rate monitoring software, Woodbury, NY, USA). Rating of perceived effort (RPE) was measured prior to first and second combats, and at the end of each round using a 15-point Borg scale [21]. The participants were instructed to incorporate both muscular and central cardio-respiratory effort into an overall assessment of perception of effort.

### 2.6. Video Data Analyses

The recorded videos were analyzed using Sony Vegas Pro 8.0^®^ software (Sony Creative Software, Middleton, WI, USA), as previously described [16]. The attack time, attack numbers, scores, skipping time, and pause time, were computed separately for each round. The attack time was considered the elapsed time from the beginning of a foot or hand movement in the direction of the opponent to when the athlete stopped this attack movement or until the time that the athlete was unable to continue attacking due to a fall or a referee-induced pause. The skipping time was defined as the total time in which there was no attempt to attack, and pause time was characterized as the time-outs enforced by the referee. The scores were attributed by the referee and recorded by the main research in a scoreboard visible to the athletes, as in a real competition.

A single investigator, who was highly experienced in, and familiar with, taekwondo matches, analyzed all videos. The evaluator was blinded to the athlete supplements. This analysis adhered to the same procedures previously used in other studies of combat sports [15,16,18]. Such an analysis was reported to be both objective and reliable, as it is used to determine the score with an intra-class correlation coefficient (ICC) greater than 0.93 [22]. Similar analysis performed by the same investigator in our previous study [16] revealed to be highly reliable for all parameters investigated, with ICC values ranging from 0.71 to 0.94 (*p* < 0.05).

### 2.7. Statistical Analyses

The sample size was estimated as recommended by Hopkins [23] using data from our previous study [16]. The data distribution was verified using a Shapiro-Wilk test. A non-normal distribution was noted for the data from the video analyses. Thus, a Wilcoxon test was used to determine whether there were differences between the conditions (caffeine and placebo), combats (combat 1 and combat 2) or rounds (1, 2, and 3). A normal distribution was observed for reaction-time tests, total scores, lactate, RPE, and HR; thus, paired t tests were used to compare between conditions (caffeine and placebo). Analyses of variance (ANOVA) with repeated measurements were used to compare these variables at each time period. Where necessary, Bonferroni multiple comparison adjustments were performed. All analyses were performed using SPSS software (version 13.0; IBM, Chicago, IL, USA). The level of significance was set to *P* < 0.05.

## 3. Results

### 3.1. Success of the Blinding and Pre-Test Recommendations

From the ten athletes investigated, none presented any abnormality in electrocardiogram. Only one athlete distinguished correctly which capsule had been ingested. In addition, participants reported no side effects of caffeine such as insomnia, nervousness and restlessness, stomach irritation, nausea and vomiting, headache, anxiety, and agitation. Furthermore, all athletes reported following pre-test instructions and confirmed that they did not drink alcohol, caffeine, and performed vigorous exercise during the 48 h before the test.

### 3.2. Reaction-Time Test

The reaction time prior to the first combat was significantly lower (−11.9%, *P* = 0.004) in athletes who had ingested caffeine *versus* placebo, but this difference was no longer significant after the first and second combats (*P* = 0.58 and 0.26, respectively, Table 1). The kick time was similar between conditions for all time points (*P* > 0.05).

**Table 1:** Mean ± standard deviation for reaction and kick times for pre-combat 1 and post-combat 1 and 2 following caffeine or placebo ingestion.

Moment	Variable (s)	Caffeine	Placebo
Pre-combat 1	Reaction time	0.37 ± 0.07 *	0.42 ± 0.05
	Kick time	0.35 ± 0.07	0.32 ± 0.06
Post-combat 1	Reaction time	0.39 ± 0.06	0.41 ± 0.06
	Kick time	0.33 ± 0.08	0.32 ± 0.03
Post-combat 2	Reaction time	0.39 ± 0.07	0.42 ± 0.09
	Kick time	0.34 ± 0.05	0.28 ± 0.05

* Significantly different from placebo (*P* = 0.004).

### 3.3. Time-Motion Analyses

#### 3.3.1. Combat 1

During round 1, the sum of time-outs enforced by the referee was lower in athletes who had ingested caffeine compared with placebo (20.83 ± 14.75 *vs.* 47.21 ± 26.32 s, respectively, *P* = 0.036). During round 2, the sum of time-outs enforced by the referee was also lower following ingestion of caffeine compared with placebo (57.45 ± 57.17 *vs.* 93.38 ± 72.57 s, respectively, *P* = 0.005). There was no significant difference between the conditions for any index in round 3 (*P* > 0.05).

When data from athletes who had consumed caffeine were compared among rounds, the attack numbers (*P* = 0.007 and *P* = 0.021), sum of attack numbers (*P* = 0.013 and *P* = 0.017), and sum of skipping time (*P* = 0.047 and *P* = 0.047) were all higher in round 1 than in rounds 2 and 3, respectively. The sum of attack numbers was also higher in round 2 than in round 3 (*P* = 0.047). Additionally, the referee time-outs values were lower in round 1 than in rounds 2 and 3 (*P* = 0.012 and *P* = 0.005, respectively). In contrast, there were no differences between the rounds for any index in the placebo trial (*P* > 0.05).

#### 3.3.2. Combat 2

During round 1, mean skipping time (6.97 ± 2.97 *vs.* 10.60 ± 5.06 s, *P* = 0.012) and the sum of skipping time (77.62 ± 19.38 *vs.* 91.71 ± 15.29 s, *P* = 0.017) were lower following caffeine consumption compared with placebo. Consequently, the attack/skipping ratio (0.15 ± 0.09 *vs.* 0.09 ± 0.05 units, respectively, *P* = 0.018) was higher following caffeine consumption compared to placebo. For round 2, the attack numbers (11.80 ± 5.41 *vs.* 9.75 ± 4.06 times, *P* = 0.028) was also higher following caffeine ingestion. There was no difference between conditions for any index in round 3 (*P* > 0.05). However, the mean of the entire combat 2 for referee time-outs (17.98 ± 5.55 *vs.* 11.38 ± 5.05 s, respectively, *P* = 0.049) and attack numbers (12.20 ± 6.71 *vs.* 8.88 ± 3.21 times, respectively, *P* = 0.035) were higher following caffeine ingestion compared with placebo.

When data were compared among rounds, differences were found only for the placebo condition. The sum of referee time-outs in round 1 was lower than in round 2 (*P* = 0.012), whereas skipping time was higher in round 1 than in round 2 (*P* = 0.012). Consequently, the attack/skipping ratio was lower in round 1 than in round 2 (*P* = 0.012). The attack numbers (*P* = 0.007) and sum of skipping time (*P* = 0.012) were lower in round 1 than in round 3.

#### 3.3.3. Combat 1 *versus* Combat 2

**Figure 2:**
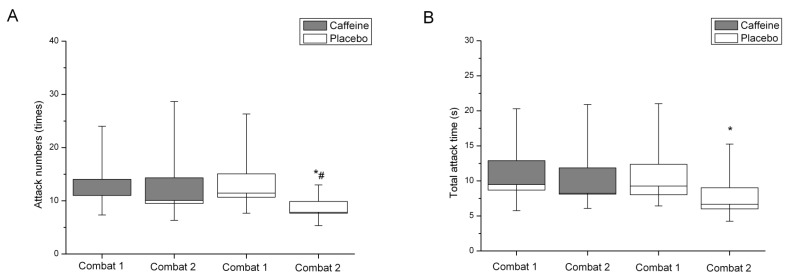
Box and whisker plot of attack numbers (**A**) and total attack time (**B**) during the first and second combats following caffeine and placebo supplementation. * Significantly lower than combat 1 for the same condition. # Significantly lower than caffeine within the same combat. Data are of the median, standard error and 95% confidence interval.

There were no differences in mean combat indices between combats 1 and 2 when caffeine was consumed (*P* > 0.05). In contrast, attack numbers (*P* = 0.035) and the total attack time (*P* = 0.036) were lower in combat 2 than in combat 1 when athletes consumed placebo (Figure 2). Moreover, in the placebo trial, the relative attack time was lower (*P* = 0.025) whereas skipping time was higher (*P* = 0.049) in combat 2 than in combat 1.

### 3.4. Plasma Lactate, Heart Rate, and RPE

Plasma lactate concentration increased progressively in both conditions and over combats (*P* < 0.001, Table 2). However, the values were higher following caffeine ingestion compared with placebo in rounds 2 (*P* = 0.029) and 3 (*P* = 0.014) during the first combat. Nevertheless, RPE (Table 2) and HR (Table 3) patterns were similar between conditions during both combats (*P* > 0.05).

**Table 2:** Blood lactate and ratings of perceived exertion (RPE) values (mean ± SD) for rounds 1, 2, and 3 of combats 1 and 2 following caffeine or placebo ingestion.

Moment	Lactate (mmol·L^−1^)		RPE (units)	
	Caffeine	Placebo	Caffeine	Placebo
Pre-combat 1	1.3 ± 0.5	1.5 ± 1.2	7.3 ± 1.1	8.4 ± 1.4
Round 1	5.7 ± 2.4	4.8 ± 1.3 ^#^	10.9 ± 1.6	11.1 ± 2.6
Round 2	10.4 ± 5.2 ^#,^*	7.9 ± 2.5 ^#^	12.6 ± 2.6 ^#^	12.6 ± 2.8 ^#^
Round 3	12.6 ± 6.2 ^#,^*	9.3 ± 2.8 ^#,§^	13.3 ± 3.4 ^#^	12.8 ± 3.2 ^#^
Pre-combat 2	3.2 ± 1.9	2.3 ± 0.7	8.0 ± 1.6	8.9 ± 1.4
Round 1	5.8 ± 2.8	5.1 ± 2.0	11.1 ± 2.8	10.6 ± 2.1
Round 2	7.6 ± 3.6	6.4 ± 2.8 ^#^	11.0 ± 4.4	11.6 ± 2.0
Round 3	10.0 ± 5.3 ^#^	8.0 ±3.0 ^#^	14.5 ± 3.1 ^#^	12.5 ± 3.0

* Significantly different from placebo. ^#^ Significantly different from pre-combat within the same condition. ^§^ Significantly different from round 1 within the same condition and same combat.

**Table 3:** Heart rate (peak and mean) values (mean ± SD) during rounds 1, 2, and 3 of combats 1 and 2 following caffeine or placebo ingestion.

Moment		Peak (bpm)		Mean (bpm)	
		Caffeine	Placebo	Caffeine	Placebo
Combat 1	Round 1	205 ± 24	202 ± 32	169 ± 15	161 ± 21
	Round 2	208 ± 25	204 ± 35	168± 10	147 ± 52
	Round 3	200 ± 40	192 ± 25	168 ± 20	166 ± 18
Combat 2	Round 1	186 ± 33	197 ± 24	154 ±30	160 ± 18
	Round 2	201 ± 23	203 ± 34	168 ± 20	168 ± 13
	Round 3	208 ± 27	191 ± 33	170 ± 19	172 ± 24

## 4. Discussion

The main findings of this study were that caffeine ingestion (1) improved reaction time prior to the first combat; (2) increased the intensity of round 1 of the first combat, thereby may have resulted in higher plasma lactate concentrations after round 2 and 3 in the first combat; and (3) maintained the intensity of the second combat at a level similar to that of the first combat, whereas intensity was reduced following placebo ingestion.

In this study, time taken to respond to a visual stimulus prior to the first combat was faster when supplemented with caffeine. In a previous study, Gillingham *et al.* [4] reported that caffeine decreased the time spent for a shooter to identify a target and make the shot. Souissi *et al.* [24] verified that the time taken to answer a visual stimulus by pushing a key in a microcomputer was lower following caffeine ingestion compared with placebo in elite judo athletes. This effect of caffeine during the initial reaction time test may be related to an enhanced arousal since caffeine seems to promote arousal by activating pathways that traditionally have been associated with motivational and motor responses in the brain [25]. On the other hand, although this was not significant, reaction time was consistently faster and kick time consistently slower with caffeine. This result is in accordance with Gillingham *et al.* [4] whom suggested that caffeine ingestion improves target detection and engagement speed during vigilance situations, but may not effective during more complex operations requiring higher levels of cognitive processing and fine motor control and coordination. However, to the best of our knowledge, this is the first study to show that caffeine ingestion improves reaction time, but not kick time, during a sport-specific Taekwondo test. Nevertheless, we also found that the effects of caffeine on reaction time disappeared after the first and second combats. The reasons underlying this effect remain unclear; however, they may be related to the increase in adenosine levels during the exercise [2,17,26]. An increase in adenosine levels may lead to competition between adenosine and caffeine for adenosine receptors in the CNS [27]. This higher competition for adenosine receptors may reduce the effects of caffeine on neuronal excitability. Alternatively, this effect may have resulted from a postactivation potentiation effect on reaction time after the combats [28,29]. It has been demonstrated that when performing any exercise prior to a main task, the performance can improve during the main task due a reduced firing threshold of the motoneurons, suggesting that the effects of caffeine on reaction time may “overlap” any postactivation potentiation effect in our study [28,29]. However, because the time to react did not improve between pre- and post-combat one and two in either condition, the effect of postactivation potentiation is likely small.

Video analyses revealed that there were a lower number of referee break times following ingestion of caffeine compared with placebo during rounds 1 and 2 of the first combat. These data suggest that athletes may have concentrated more during the combat, thereby reducing penalties. As this finding was accompanied by higher lactate values, combat was intensified slightly during the first combat, which corroborates studies that examined the effect of caffeine on repeated sprints. For example, Lee *et al.* [30] reported that caffeine increased peak power output during the first two sprints of a set of 6 × 10-s sprints. Similarly, Glaister *et al.* [10] also demonstrated an improved time during the 3 first sprints of a set of sprints (12 × 30 m; 35-s intervals) following ingestion of caffeine. The higher plasma lactate level in the first combat in our study suggests more intense glycolytic anaerobic system activation, and that the additional energy required supporting a more intense combat must have been offered by this system. These findings corroborates previous studies using intermittent exercises in which blood lactate levels were higher in the first sprints when athletes were supplemented with caffeine [10,12,30]. On the other hand, Matsushigue *et al.* [15] investigated Songahm Taekwondo and concluded that glycolytic metabolism was not the predominant energy source. We found an elevated plasma lactate concentration after the round 3 in both the combats (8–9 mmol·L^−1^), and caffeine increased plasma lactate considerably (10–12 mmol·L^−1^). These results suggest that, different from Songahm Taekwondo style, WTF Taekwondo style has an important glycolytic contribution and that can be increased by caffeine intake.

Another interesting result from this study was that time-motion parameters analyzed were similar between combat 1 and 2 following caffeine consumption but that the total attack time were reduced and skipping time was increased following placebo consumption. These data suggest that caffeine enabled maintenance of intensity during both combats. In addition, during the first rounds of the second combat, parameters related to recovery (mean and summed skipping times) were lower, whereas parameters related to effort (attack numbers and attack/skipping ratio) were higher following caffeine ingestion compared with placebo. Caffeine seems to increase anxious arousal, impulsiveness and risk-taking [31]. There is also some evidence that caffeine consumption can be associated with changes in the hormone testosterone which is related to aggression and an increase in risk-taking behaviors [32]. Together, these findings suggest that athletes may have unconsciously taken an increased risk, increasing therefore the attack numbers. Despite these results, RPE and HR responses were similar between conditions, suggesting that although athlete intensities were higher during the second combat following ingestion of caffeine this intensity was performed with a similar perceived effort compared with placebo ingestion. Thus, the perception of efforts after caffeine consumption was less than actual efforts. These results are supported by findings showing an increased intensity of effort during the final part of an intermittent running-sprints protocol (6 × 15 m) [9] and simulated tennis match [33] after caffeine ingestion without a concomitant increase in RPE. Thus, our results suggest that coaches and trainers should consider caffeine supplementation to enable athletes to increase the intensity of combat. In particular, when athletes fight several times within a single day, as is common in official championships, caffeine supplementation may delay fatigue and improve athletic performance. Despite in the present study we have not investigated the chronic effect of caffeine it is plausible to suspect that caffeine ingestion may also increase the intensity of training sessions, increasing training adaptations.

Our results demonstrate a delay in fatigue following caffeine consumption. It should be noted that the more intense combat was not associated with a significantly higher number of points scored, although the mean scores were consistently higher following caffeine ingestion compared with placebo throughout the second combat. It may be an indicative that caffeine could result in some extra points in a combat, implicating in a slightly improved performance that may have a practical significance in the real competition world. It should also be mentioned that combats were performed between supplemented athletes, *i.e.*, caffeine *versus* caffeine. Further studies combining supplemented *versus* non-supplemented athletes may provide novel insights into the effects of caffeine on performance during taekwondo combats.

This study has some relevant limitations. It is important to note that time-motion analyses may involve subjective perceptions and decisions of the investigator. This technique has been demonstrated to be highly objective and reliable [16], and in this study, the same experienced investigator who was blinded to supplement (caffeine or placebo) evaluated all combats; however, any error derived from video analyses cannot be fully disregarded. In addition, further studies should measure the reproducibility of the caffeine effects on parameters derived from video analyses. Furthermore, because there is a degree of variability in the magnitude of response to caffeine ingestion, due in part to a single nucleotide polymorphism influencing caffeine metabolism and outcomes from caffeine ingestion [34], further studies should control the effects of caffeine on Taekwondo performance in responder and non-responders.

In summary, considering clinical practice and practical applications, our findings suggest that: (1) Caffeine supplementation may be used to improve reaction times in taekwondo athletes; (2) Coaches and trainers should consider caffeine supplementation to enable athletes to increase the intensity of combat; (3) When athletes fight several times within a single day, caffeine supplementation may delay fatigue and improve athletic performance, and (4) Caffeine ingestion may also increase the intensity of training sessions.

## 5. Conclusions

The ingestion of an acute, moderate dose of caffeine (5 mg·kg^−1^ body mass) reduced reaction time during a sport-specific task in a non-fatigued state and shorted stop times (referee break times) during the early rounds of a simulated combat. Intensity was also greater during the first rounds of the second combat following caffeine consumption, whereas intensity of the entire second combat was similar to the first, suggesting that caffeine may delay fatigue during successive taekwondo combats.
